# Supplementary figures and images for: Dietary Cottonseed Protein Substituting Fish Meal Induces Hepatic Ferroptosis Through SIRT1-YAP-TRFC Axis in Micropterus salmoides: Implications for Inflammatory Regulation and Liver Health
Source: Biology (Basel). 2025 Jun 23;14(7):748. doi: 10.3390/biology14070748 (PMC12292115; doi:10.3390/biology14070748)

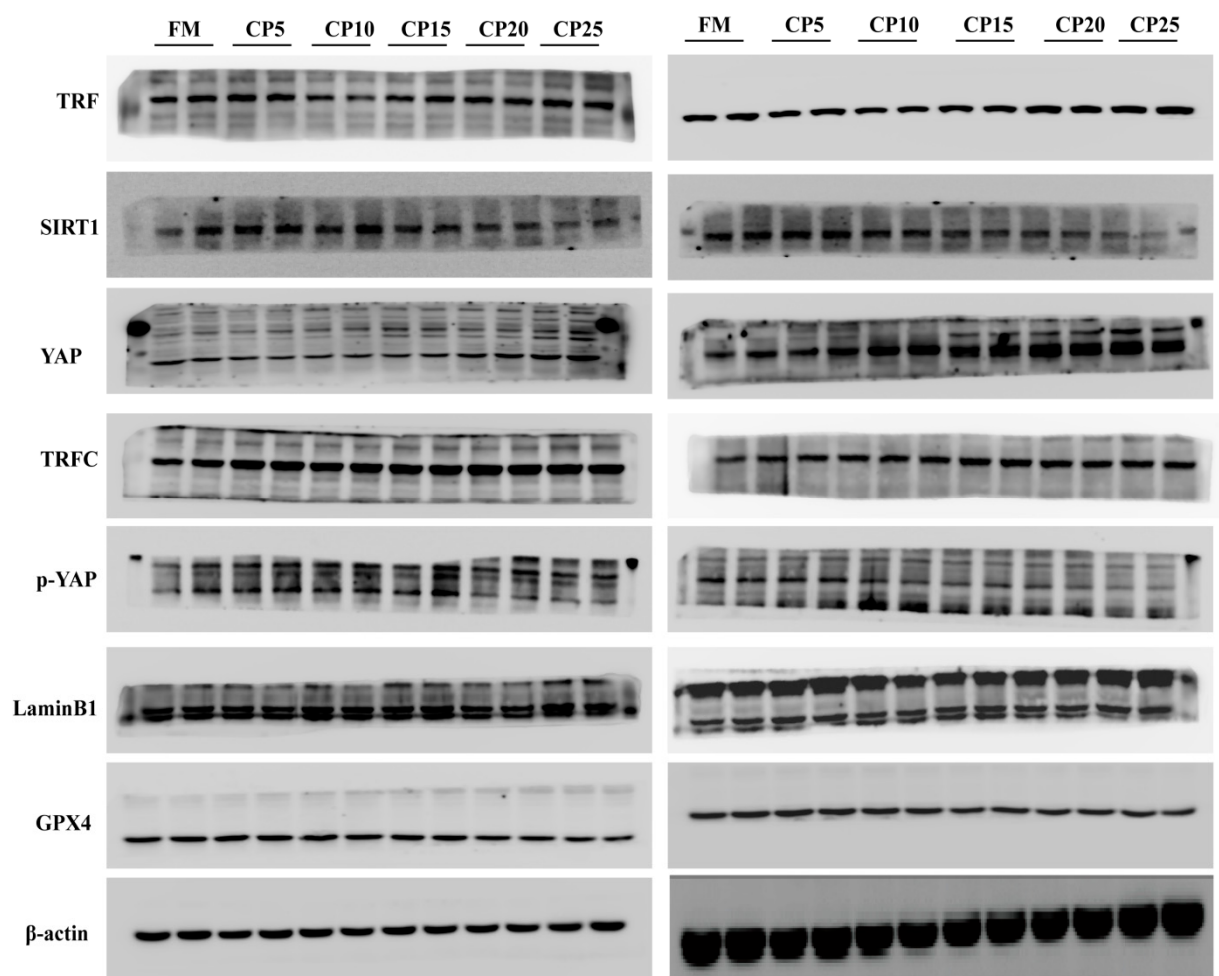

Figure S1 Western Blot Original Images.

Supplement: Supplementary file 1 [file biology-14-00748-s001.zip › biology-3597485-Supplementary Materials.pdf]
